# The weaker sex? What we can learn from sex differences in population mental health during and beyond the COVID-19 pandemic

**DOI:** 10.1007/s00406-021-01312-5

**Published:** 2021-07-29

**Authors:** Katrin Elisabeth Giel, Birgit Derntl

**Affiliations:** 1 Department of Psychosomatic Medicine and Psychotherapy, Medical University Hospital Tübingen, Osianderstr. 5, 72076 Tübingen, Germany; 2 University Hospital for Psychiatry and Psychotherapy, Calwerstr. 14, 72076 Tübingen, Germany

Since the outbreak of the COVID-19 pandemic, there has been heightened attention towards population mental health due to the pandemic-related stress induced by profoundly changed life circumstances with recurrent lockdown situations worldwide. Representative data assessed early after the outbreak of COVID-19 show an overall increase in mental distress in the general population, and they also support that population subgroups are affected differently [1, 2]. There is up to now overwhelming evidence from both, large representative population-based surveys as well as data from convenience samples from nearly all regions of the globe, that the COVID-19 pandemic affects mental health and well-being of men and women differently [3]. Overall, data on the effects of the pandemic in early stages on population mental health seem to identify females as the more vulnerable sex [3]. For instance, women were more likely to report higher levels of depression and anxiety [1, 2] and severe experience of loneliness during lockdown conditions [4].

Such findings on sex differences of the adverse effects of the COVID-19 pandemic are pivotal to inform policy decisions as well as prevention and health service strategies. However, it might be worthwhile to take a step back and reflect on this potential sex difference, especially as this is rarely discussed in the original research papers. Firstly, is it really the case that females are more vulnerable to experience mental health deterioration under the pandemic? And if yes, what are the underlying causes of this sex difference?

Regarding the first question, it is important to consider common methods of mental health assessment: most studies focus on symptoms of anxiety and depression and do rely on screening or brief self-report instruments [3]. While this makes a lot of sense as anxiety and mood disorders are the most prevalent mental health issue in the general population, it also poses the challenge that anxiety and depression are often expressed differently in women versus men and that men might be less likely to agree to the items used in these common assessment instruments. These reporting differences might partly be rooted in traditional societal gender roles which “permit” rather females than males to feel anxious or depressed. Hence, the apparent sex differences in mental health outcomes during the pandemic might at least partly draw back on assessment and reporting biases, and they would potentially be shifted if different instruments would be used. Secondly, chronic stress might lead to different mental health outcomes in men than in women, for instance, men might suffer less often from internalizing problems such as anxiety and depression, but more often from externalizing problems such as substance use. Indeed, recent evidence from large-scale health care use data shows that men were more likely to be admitted to an US emergency department due to drug overdose during the COVID-19 pandemic [5]. However, most large-scale population-based studies do not ask for mental health issues beyond the most prevalent symptom domains, probably resulting in under-detection of mental health issues in males.

Regarding the second question, it is important to consider potential underlying causes of women tending to report higher mental health burden in surveys. Some of them might lie outside biological sex differences and might again draw back on societal gender roles and socioeconomic differences between men and women. While there is substantial evidence indicating that women report greater fear and anxiety, this may be due to a broad range of biological influences, temperamental and cognitive factors, as well as socialization processes. A large representative survey identified people caring for pre-school children as vulnerable subgroup experiencing elevated distress early after COVID-19 outbreak [1]. In communities predominantly allocating care work to women, difficulties associated with childcare shutdowns during the pandemic might result in female survey participants with small children reporting elevated levels of distress, which is mainly due to their gender role and not to their biological sex.

Moreover, most studies that report sex differences rely on more data from females than males, particularly when self-report measures or experience sampling methods were applied [3].

Adverse life events are considered primary sources of risk for negative mental health outcomes including mental disorders, however, most individuals withstand, adapt to and facilitate recovery from adversity. Sex differences in resilience to stress have been reported, though most experimental work has been done in animals, indicating that while the behavioral outcome may be similar, the underlying neural circuits and operating resilience factors may not be concordant between the sexes [6]. For example, exercise-induced stress resistance via physical activity is one aspect where both, females and males, benefit in terms of stress reduction and depression, with females potentially responding faster [6]. Furthermore, many individuals who experience mental health issues following stress fully recover later on, highlighting the complexity of risk and resilience in humans.

In line with this, more recent longitudinal studies and meta-analyses draw a more differential picture regarding population mental health during COVID-19, indicating that a vast majority of people is resilient [7, 8]. Of note, high-quality data from representative surveys modelling longitudinal mental health trajectories do not identify sex differences any longer [8–10] or outlines that females who initially reacted with increased levels of anxiety and depression rapidly adapted to the situation and belonged to the groups with fastest mental health improvements in the first weeks after lockdown [9]. This outlines the importance to differentiate between acute and long-term reactions to (pandemic-related) stress and their interactions with potential sex differences.

The global pandemic can be positively used for mental health research and practice if it motivates us to work towards a more sex- and gender-sensitive research on mental health outcomes which considers and overcomes potential biases, including stereotypical and binary concepts, and at the same time, does not overlook specific needs of all included groups. This would ultimately also result in a better detection and recognition of mental health needs of and within the studied groups as many other factors interacting with biological sex and gender role will be assessed. By striving for this, we can improve our prevention and treatment efforts for the whole society.
